# Creatine kinase B mediates UCP1-independent beige fat thermogenesis via the futile creatine cycle in mice

**DOI:** 10.1016/j.molmet.2025.102193

**Published:** 2025-06-23

**Authors:** Jakub Bunk, Mina Ersin, Mohammed F. Hussain, Bozena Samborska, Maria Guerra-Martinez, Drishti Soni, Lawrence Kazak

**Affiliations:** 1 Rosalind & Morris Goodman Cancer Institute, McGill University, Montreal, QC, H3A 1A3, Canada; 2 Department of Biochemistry, McGill University, Montreal, QC, H3G 1Y6, Canada

**Keywords:** UCP1-indpendent thermogenesis, Futile Creatine Cycle, Creatine Kinase B, Tissue-nonspecific alkaline phosphatase, Beige adipocytes, Isotope tracing, Inguinal white adipose tissue, Interscapular brown adipose tissue

## Abstract

**Objectives:**

Creatine kinase B (CKB) is the main isoenzyme driving creatine kinase (CK) activity in classical brown adipocytes. However, the specific CK isoenzyme active in beige adipocytes remains unknown. This study aimed to identify the predominant CK isoenzyme expressed and functionally active in beige adipocytes.

**Methods:**

CK activity was tracked using D3-creatine tracing in inguinal adipocytes from mice with adipocyte-specific *Ckb* deletion and their littermate controls, across *in vivo* and *in vitro* settings.

**Results:**

CKB was essential for CK activity in protein lysates and intact white and beige adipocytes isolated from inguinal fat and drives thermogenesis through the Futile Creatine Cycle.

**Conclusions:**

Similar to classical brown adipocytes, CKB is the key functional CK isoenzyme in white and beige adipocytes from the inguinal fat depot.

## Highlights

• Adipocytes take up extracellular creatine and convert it to phosphocreatine *in vivo.*
• CKB mediates creatine kinase activity in white and beige adipocytes.
• CKB is essential for beige adipocyte thermogenesis through the Futile Creatine Cycle (FCC).
• FCC activity in beige fat mitochondria is CKB- and TNAP-dependent.

## 1 Introduction

Adipocyte thermogenesis dissipates macronutrient energy as heat and is driven via uncoupling macronutrient oxidation from ATP-synthase activity through uncoupling protein 1 (UCP1) and UCP1-independent futile cycles that promote ATP synthesis and turnover [1, 2, 3, 4, 5, 6, 7, 8, 9, 10, 11, 12, 13]. Under conditions of low adrenergic stimulation, such as standard room temperature and thermoneutral housing, thermogenesis in subcutaneous white adipocytes is minimal. However, cold exposure triggers adaptive tissue, cell and organelle remodeling, giving rise to thermogenic beige adipocytes, which can be derived from precursors that are distinct to classical brown adipocytes [14, 15, 16, 17, 18]. When activated, beige adipocytes, like brown adipocytes, can drive macronutrient burning through the actions of UCP1 and UCP1-independent mechanisms [1,2,6,12,13,19, 20, 21, 22].

Creatine kinases (CKs) traditionally regulate cellular energy homeostasis in cells with high and fluctuating energy demands via the phosphocreatine circuit. This process mediates the transphosphorylation of phosphate from mitochondrial or glycolytic ATP to creatine to generate phosphocreatine, which is then used by cytosolic CK to maintain the free energy of ATP hydrolysis [23, 24, 25]. In most cells, phosphocreatine–creatine phosphotransfer occurs in a 1:1 ratio with ATP–ADP. However, in thermogenic adipocytes, creatine stimulates a mitochondrial-localized substrate cycle that liberates excess ADP. This Futile Creatine Cycle (FCC) involves a cycle of phosphocreatine synthesis and hydrolysis which triggers ATP turnover, oxygen consumption and thermogenesis [20]. In brown fat, the FCC is mediated by creatine kinase b (CKB) and tissue-nonspecific alkaline phosphatase (TNAP) [4,26], contributing a substantial portion of total thermogenesis in a UCP1-independent manner [3,5,8]. The FCC was initially discovered in purified mitochondria from beige adipose tissue, isolated from the inguinal depot of mice that had been cold exposed for one week [20]. Yet, while TNAP has been demonstrated to play a role [26], the essential CK isoenzyme that drives the FCC in beige fat has not been determined. Here, we reveal that CKB is the key CK isoenzyme in beige adipocytes, making it essential for FCC function in these cells.

## 2 Results

### 2.1 Phosphocreatine production is impaired in iWAT and iBAT of mice lacking adipocyte CKB

**Figure 1:**
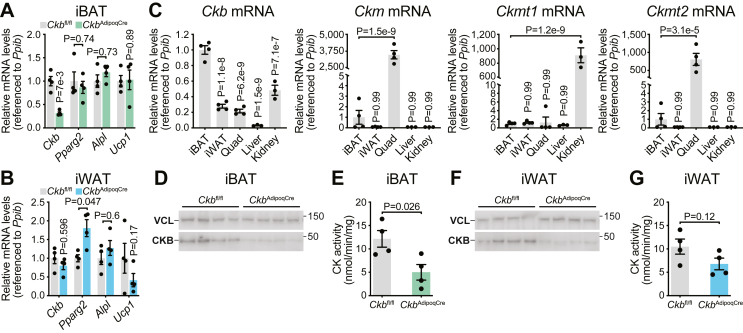
**Creatine Kinase Activity is impaired in iBAT and iWAT of mice lacking adipocyte CKB. A-G,** Intrascapular brown adipose tissue (iBAT), Inguinal white adipose tissue (iWAT), quadriceps muscle (Quad), liver and kidney were harvested from 16-week-old *Ckb*^fl/fl^ and *Ckb*^AdipoqCre^ male mice (*n* = 4 per group). **A,** RT-qPCR in iBAT. **B,** RT-qPCR in iWAT. **C,** RT-qPCR of Creatine Kinase (CK) isoenzymes in iBAT, iWAT, Quad, Liver and Kidney. **D,** Western blot of CKB in iBAT. VCL was used as a loading control. **E,** CK activity in bulk iBAT lysates. **F,** Western blot of CKB in iWAT. VCL was used as a loading control. **G,** CK activity in bulk iWAT lysates. Data are presented as mean ± s.e.m. and *n* numbers are of biologically independent experiments. *P* values were calculated using two-way ANOVA with Holm-Šídák's multiple comparisons test (**A, B**); one-way ANOVA with Holm-Šídák's multiple comparisons test (**C**), and unpaired two-tailed t-test (**E, G**).

To determine whether CKB is essential for creatine kinase (CK) activity in inguinal white adipose tissue (iWAT), we used *Ckb*^AdipoqCre^ mice, in which *Ckb* is specifically deleted in adipocytes [4]. In these mice, *Ckb* mRNA levels were reduced by about 70% in bulk interscapular brown adipose tissue (iBAT) compared to *Ckb*^fl/fl^ littermate controls (Fig. 1A), confirming efficient gene deletion, consistent with our prior work [4]. In contrast, *Ckb* mRNA levels were only modestly reduced by ∼20% within bulk iWAT of *Ckb*^AdipoqCre^ mice compared to *Ckb*^fl/fl^ littermate controls (Fig. 1B), also as we had previously demonstrated [4]. We attribute this limited reduction to the presence of *Ckb* expression in non-adipocyte (non-parenchymal) cells within iWAT, reflecting the tissue's cellular heterogeneity [27]. To further characterize CK isoform expression, we profiled all the CK isoforms (*Ckb, Ckm, Ckmt1,* and *Ckmt2*) across multiple tissues. *Ckb* mRNA was enriched in iBAT relative to iWAT, quadriceps muscle, liver, and kidney (Fig. 1C). In contrast, the other CK isoforms showed minimal expression in both iBAT and iWAT but were clearly expressed in non-adipose tissues - *Ckm* and *Ckmt2* in quadriceps and *Ckmt1* in kidney (Fig. 1C).

**Figure 2:**
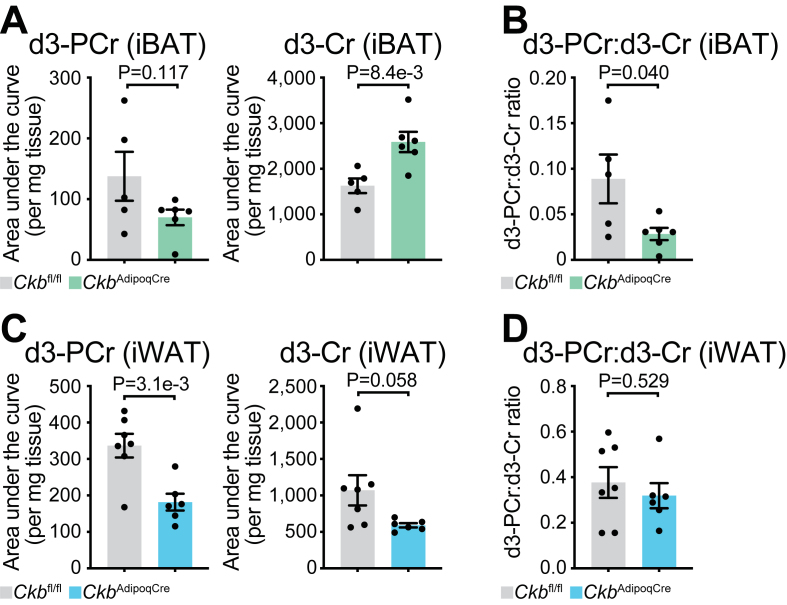
**Phosphocreatine production is impaired in iWAT and iBAT of mice lacking adipocyte CKB. A-D,** 16-week-old *Ckb*^fl/fl^ and *Ckb*^AdipoqCre^ male mice were injected intraperitoneally with deuterated (m+3) creatine (4 mg/kg), and iBAT and iWAT were harvested 4 h later. **A,** LC-MS analysis of deuterated m+3 phosphocreatine (d3-PCr; left) and deuterated m+3 creatine (d3-Cr, right) levels in iBAT of *Ckb*^fl/fl^ and *Ckb*^AdipoqCre^ male mice (*n* = 5, 6 per group). **B,** Ratio of d3-PCr to d3-Cr from Fig 2A. **C,** LC-MS analysis of d3-PCr (left) and d3-Cr (right) levels in iWAT of *Ckb*^fl/fl^ and *Ckb*^AdipoqCre^ male mice (*n* = 7, 6 per group). **D,** Ratio of d3-PCr to d3-Cr from Fig 2C. Data are presented as mean ± s.e.m. and *n* numbers are of biologically independent experiments. *P* values were calculated using unpaired two-tailed t-test (**A-D**).

We next examined CKB protein levels and CK enzymatic activity in iBAT and iWAT. In iBAT, CKB protein was markedly reduced in *Ckb*^AdipoqCre^ mice compared to *Ckb*^fl/fl^ littermate controls (Fig. 1D), resulting in an approximate 60% decrease in total CK activity (Fig. 1E). In iWAT, CKB protein levels were also lower in *Ckb*^AdipoqCre^ mice compared to *Ckb*^fl/fl^ controls (Fig. 1F); however, the reduction in total CK activity was more modest (35%) (Fig. 1G), suggesting a larger contribution from non-parenchymal cells within iWAT or a contribution of alternative CK isoforms within inguinal white adipocytes. Next, we assessed CK activity in iBAT and iWAT *in vivo* using stable-isotope tracing combined with liquid chromatography–mass spectrometry (LC–MS). Mice were intraperitoneally (i.p.) injected with a bolus of M+3 deuterated creatine (d3-creatine, 4 mg/kg), a dose previously shown to increase its phosphorylated form, d3-phosphocreatine, in muscle [28]. This approach led to an accumulation of both d3-creatine and d3-phosphocreatine in iBAT and iWAT (Supplementary Fig. 1A and 1B). We then compared *Ckb*^fl/fl^ and *Ckb*^AdipoqCre^ mice 4 h following d3-creatine injection. In iBAT, d3-phosphocreatine levels were significantly reduced, by approximately 50%, in *Ckb*^AdipoqCre^ mice relative to controls, despite a ∼60% increase in d3-creatine accumulation (Fig. 2A). Consequently, the d3-phosphocreatine to d3-creatine ratio was significantly lower in iBAT from *Ckb*^AdipoqCre^ mice compared to *Ckb*^fl/fl^ controls (Fig. 2B), indicating impaired CKB-mediated phosphorylation. This suggests that CKB activity is primarily localized within parenchymal adipocytes rather than stromal vascular cells in iBAT. In contrast, although d3-phosphocreatine levels in iWAT were also reduced by ∼46% in *Ckb*^AdipoqCre^ mice, this mirrored a proportional reduction in intracellular d3-creatine levels (Fig. 2C), resulting in no significant difference in the d3-phosphocreatine:d3-creatine ratio (Fig. 2D). Levels of endogenous (unlabeled) phosphocreatine and creatine, as well as their ratio in iBAT were not significantly different between genotypes (Supplementary Fig. 2A and 2B). Endogenous phosphocreatine levels were lower in *Ckb*^AdipoqCre^ iWAT compared to *Ckb*^fl/fl^ controls without any difference in creatine; however, this did not alter the phosphocreatine to creatine ratio between genotypes (Supplementary Fig. 2C and 2D). Together, these findings demonstrate that CKB regulates phosphocreatine production in iBAT (as determined by d3-creatine tracing). However, we posited that due to the established cellular heterogeneity of iWAT [27], including contributions from non-adipocyte populations, the specific role of adipocyte CKB in this depot cannot be accurately assessed using bulk tissue analyses.

### 2.2 Non-parenchymal cells of white, beige, and brown fat predominantly express *Ckb*

**Figure 3:**
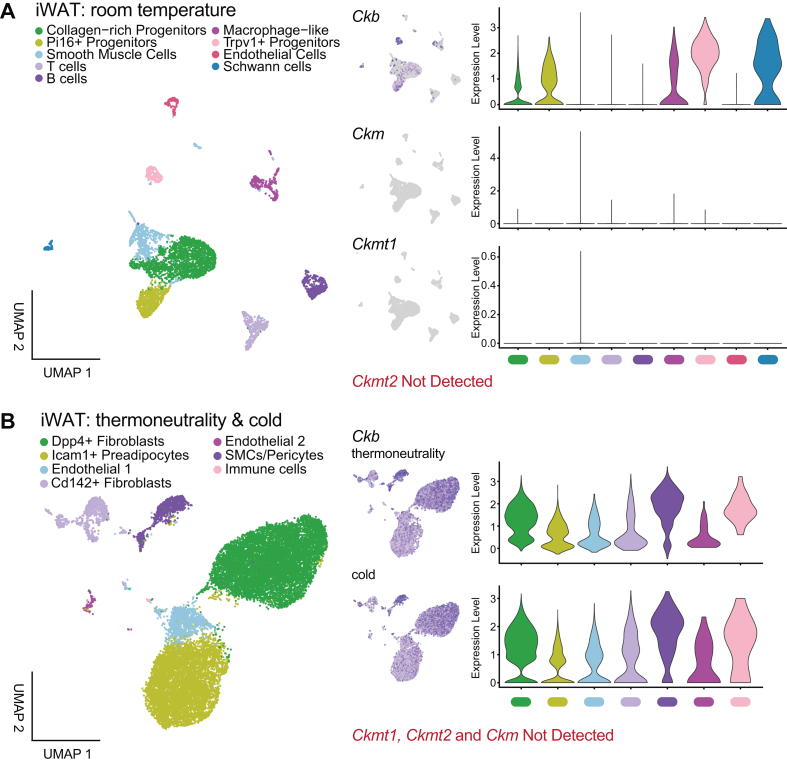
**Single cell RNA sequencing (scRNA-seq) analysis of iWAT stromal vascular cells. A,** scRNA-seq of stromal vascular cells obtained from GSE154047. Integrated UMAP plot (left) with individual gene UMAP plots (middle) and violin plots (right) showing the expression levels of detected CKs. **B,** scRNA-seq of stromal vascular cells obtained from GSE227441. Integrated UMAP plot (left) with individual gene UMAP plots (middle) and violin plots (right) showing the expression levels of detected CKs.

To examine whether non-parenchymal cells express CKs that could contribute to net CK activity in bulk adipose tissue analyses, we mined publicly available single cell RNA sequencing (scRNA-seq) data. Remarkably, this analysis revealed that *Ckb* is the predominant CK isoform expressed in non-parenchymal cells of both iWAT and iBAT across thermoneutrality, room temperature, and cold exposure (Fig. 3A and B and Supplementary Fig. 3). In iWAT at room temperature, *Ckb* expression was highest in Schwann cells and Trpv1^+^ progenitors (Fig. 3A). In a dataset of mice housed under thermoneutral and cold conditions, fibroblasts, pericytes, and immune cells emerged as the primary *Ckb*-expressing cell types in iWAT-derived stromal cells (Fig. 3B). In iBAT, vascular smooth muscle cells, immune cells and Schwann cells were the main sources of *Ckb* expression, regardless of temperature condition (Supplementary Fig. 3). In contrast, we found little evidence of *Ckmt1*, *Ckmt2*, or *Ckm* expression across all non-parenchymal cell types and temperature conditions (Fig. 3A and B and Supplementary Fig. 3). These data suggest that the modest reduction in CK activity observed in bulk iWAT from *Ckb*^AdipoqCre^ mice likely reflects residual CKB activity in non-parenchymal cells, which outnumber adipocytes in this depot.

### 2.3 CKB regulates CK activity in cultured inguinal white adipocytes

**Figure 4:**
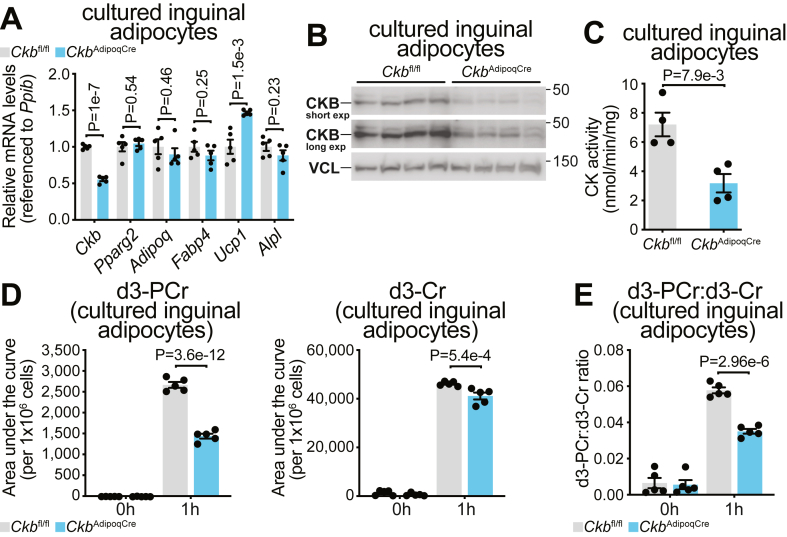
**CKB regulates CK activity in cultured inguinal white adipocytes. A-C,** Stromal vascular fraction from iWAT of 8-day-old *Ckb*^fl/fl^ and *Ckb*^*AdipoqCre*^ female mice was differentiated *in vitro* into adipocytes for 7 days. **A,** RT-qPCR of cultured inguinal adipocytes. **B,** Western blot for CKB from cultured inguinal adipocytes. VCL was used as a loading control. **C,** CK activity in cultured inguinal adipocyte lysates. **D-E,** Cultured inguinal adipocytes were incubated for 1 h with 150 μM d3-Cr (*n* = 5 per group). **D,** LC-MS analysis of d3-PCr (left) and d3-Cr (right) levels in cultured *Ckb*^fl/fl^ and *Ckb*^AdipoqCre^ inguinal adipocytes. **E,** Ratio of d3-PCr to d3-Cr from Fig 3D. Data are presented as mean ± s.e.m. and *n* numbers are of biologically independent experiments. *P* values were calculated using unpaired t-test (**A, C**), and two-way ANOVA with Holm-Šídák's multiple comparisons test (**D, E**).

To minimize the impact of cellular heterogeneity in iWAT, we isolated the stromal vascular fraction (SVF) from the iWAT of *Ckb*^AdipoqCre^ and *Ckb*^fl/fl^ mice and differentiated the preadipocytes into primary inguinal adipocytes *in vitro*. After seven days of differentiation, mRNA levels of *Pparg2*, *Adipoq*, and *Fabp4* were similar between genotypes, indicating that both groups underwent comparable adipocyte differentiation (Fig. 4A). As previously reported in iBAT [4], *Ucp1* mRNA expression was modestly, but significantly, upregulated in *Ckb*^AdipoqCre^ inguinal adipocytes compared to *Ckb*^fl/fl^ controls (Fig. 4A). Notably, *Ckb* mRNA (Fig. 4A), CKB protein levels (Fig. 4B), and total CK activity (Fig. 4C) were all significantly reduced in inguinal adipocytes from *Ckb*^AdipoqCre^ mice relative to *Ckb*^fl/fl^ controls. The reduction in CK activity closely mirrored the decrease in *Ckb* mRNA and CKB protein levels, suggesting that CKB is a major contributor to CK activity in these cells. To further assess creatine metabolism, we incubated adipocytes with d3-creatine for 1 h. Both genotypes showed comparable uptake of the labeled substrate, with *Ckb*^fl/fl^ adipocytes accumulating slightly more d3-creatine (∼10%) (Fig. 4D). Notably, *Ckb*^fl/fl^ adipocytes produced approximately 45% more d3-phosphocreatine compared to *Ckb*^AdipoqCre^ cells (Fig. 4D). This difference was further underscored by a ∼40% higher d3-phosphocreatine:d3-creatine ratio in *Ckb*^fl/fl^ cells, indicating more efficient phosphocreatine synthesis in the presence of functional CKB (Fig. 4E). The endogenous phosphocreatine:creatine ratio was significantly lower in *Ckb*^AdipoqCre^ adipocytes, while baseline levels of unlabeled creatine were similar between genotypes (Supplementary Fig. 4A and 4B). Together, these data support a role for CKB in regulating 10.13039/501100001515CK activity and phosphocreatine synthesis in cultured inguinal adipocytes. However, since our *in vitro* differentiation system did not completely remove all *Ckb* mRNA expression, these results were not conclusive. We hypothesized that the residual CK activity observed *in vitro* may arise from undifferentiated preadipocytes or immature adipocytes, likely due to the short duration and non-physiological conditions of *in vitro* differentiation. Thus, we designed additional experiments using acutely isolated mature inguinal adipocytes to resolve the specific requirement for CKB more definitively.

### 2.4 CKB is required for CK activity in mature inguinal white and beige adipocytes

**Figure 5:**
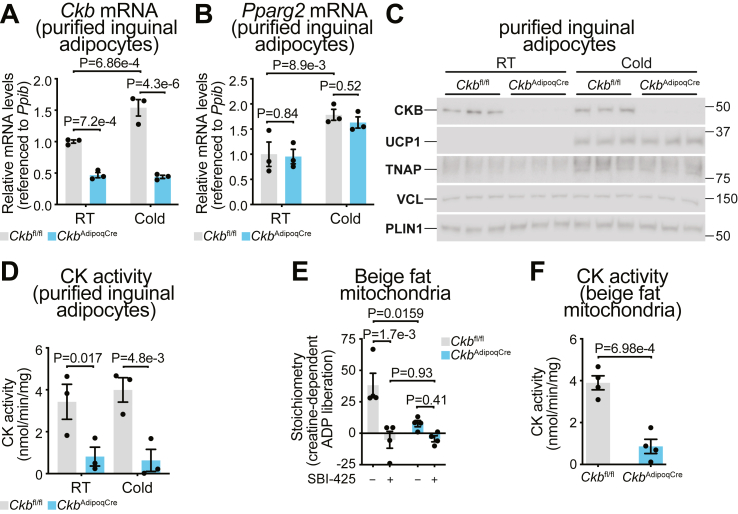
**CKB is required for CK activity in mature white adipocytesand FCC-dependent thermogenesis in beige adipocytes. A-D,** Mature adipocytes (*n* = 3 per group) were isolated from iWAT of 12–13-week-old *Ckb*^fl/fl^ and *Ckb*^AdipoqCre^ male mice exposed to RT or Cold for 3 weeks. **A,** RT-qPCR for *Ckb*, **B,** RT-qPCR for *Pparg2*, **C,** Western blot for CKB, UCP1, TNAP. VCL and PLIN1 were used as loading controls. **D,** CK activity. **E-F,** Mitochondria were isolated from iWAT of 12–13-week-old *Ckb*^fl/fl^ and *Ckb*^AdipoqCre^ male mice exposed to cold for 3 weeks (*n* = 4 per group). **E,** Stoichiometry of ADP liberation over added Cr (10 mM) as measured by respirometry. TNAP was inhibited by SBI-425 (10 mM). **F,** CK activity of beige fat mitochondrial lysates. Data are presented as mean ± s.e.m. and *n* numbers are of biologically independent experiments. *P* values were calculated using two-way ANOVA with Fisher LSD post-hoc test (**A,B,D,E**), and unpaired Two-tailed t-test (**F**).

Both *in vivo* and *in vitro* approaches highlighted a role for CKB in creatine metabolism in white and beige adipocytes, but each had limitations. *In vivo* studies preserved physiological context but included non-adipocyte contributions. *In vitro* models reduced cellular heterogeneity, allowing adipocyte-specific analysis, but showed incomplete *Ckb* deletion, likely due to residual undifferentiated cells and weak Adipoq–Cre activity during short differentiation periods. Moreover, *in vitro* systems lack the complex cues, such as cold-induced remodeling, required for beige adipogenesis, limiting their ability to fully model beige fat biology [29, 30, 31, 32, 33, 34]. To overcome the limitations of both systems, we used an *ex vivo* strategy where we isolated mature inguinal white adipocytes from *Ckb*^fl/fl^ mice acclimated to room temperature (RT; 21 °C ± 1 °C). Western blotting confirmed successful separation of adipocytes from stromal vascular cells, as indicated by enrichment of the adipocyte marker perilipin 1 (PLIN1) (Supplementary Fig. 5A). To assess CKB function under low and high thermogenic demand, *Ckb*^fl/fl^ and *Ckb*^AdipoqCre^ mice were housed at RT or cold (4 °C ± 1 °C) for three weeks. Isolated iWAT adipocytes from cold-acclimated *Ckb*^fl/fl^ mice showed significant induction of *Ckb* mRNA, whereas *Ckm*, *Ckmt1*, *and Ckmt2* were unaffected (Fig. 5A and Supplementary Fig. 5B–4D). *Ckb* mRNA expression remained low in *Ckb*^AdipoqCre^ adipocytes under both conditions (Fig. 5A), confirming efficient knockout. *Pparg2* expression was unchanged (Fig. 5B), indicating comparable adipocyte purity. Protein analysis mirrored these findings: CKB was ablated in *Ckb*^AdipoqCre^ adipocytes, and cold exposure upregulated TNAP and UCP1 (Fig. 5C), validating beige adipocyte induction and coordinated activation of the FCC- and UCP1-dependent thermogenic programs. CK activity was significantly reduced in RT-housed *Ckb*^AdipoqCre^ adipocytes and failed to increase with cold (Fig. 5D), highlighting the dependence on CKB for this activity.

### 2.5 CKB drives UCP1-independent thermogenesis by the FCC in beige adipocyte mitochondria

To define the role of CKB in UCP1-independent thermogenesis through the FCC, we revisited the observation that creatine triggers a super stoichiometric increase in oxygen consumption in mitochondria from cold-acclimated iWAT of mice [20]. We isolated mitochondria from the iWAT of cold-acclimated *Ckb*^fl/fl^ and *Ckb*^AdipoqCre^ mice (beige fat mitochondria) and measured creatine-stimulated, ADP-linked respiration. As previously observed in beige fat mitochondria of wild-type mice, creatine triggered a superstoichiometric increase in respiration in *Ckb*^fl/fl^ beige fat mitochondria, liberating ∼40-fold more ADP than expected from a single phosphocreatine synthesis event (Fig. 5E). This effect was roughly twice as strong as observed in mitochondria from cultured brown adipocytes [4]. This superstoichiometric ADP production reflects phosphocreatine hydrolysis by TNAP, which regenerates creatine and supports repeated cycles of ADP generation via CKB [35]. Consistent with this model, TNAP inhibition (SBI-425) fully blocked the response (Fig. 5E), confirming that both TNAP activity and creatine are required for FCC-driven respiration. In contrast, beige fat mitochondria from *Ckb*^AdipoqCre^ mice showed a severely blunted respiratory response and significantly reduced CK activity (Fig. 5E and F), demonstrating that CKB is essential for this process. These findings establish CKB as the key CK isoenzyme driving FCC-based thermogenesis in beige fat and highlight its tight functional coupling with TNAP.

## 3 Discussion

Although the FCC was first described in beige adipose tissue, the CK isoform responsible for driving this process, mitochondrial CKB, had previously been identified only in classical brown adipocytes, leaving its identity in beige adipocytes unresolved. While translating ribosome affinity purification (TRAP) sequencing data indicate that the canonical mitochondrial isoforms *Ckmt1* and *Ckmt2* are not expressed in parenchymal white, beige, or brown adipocytes in mice [29], the possibility remained that rare thermogenic adipocytes might express these isoforms below detection thresholds. Challenging this notion, our data show that deletion of *Ckb* in beige adipocytes leads to a marked reduction in CK activity and severely impaired FCC-driven thermogenesis, confirming that CKB, not CKMT1 or CKMT2, is the functionally relevant CK in this context. This conclusion is further supported by evidence where CK activity was significantly reduced in purified inguinal white and beige adipocytes lacking CKB. Together with earlier studies identifying CKB as essential for regulating CK activity in classical BAT [3, 4, 5, 6], our findings here expand the role of CKB as the principal isoform driving FCC-dependent thermogenesis across all major thermogenic adipocyte types in mice.

Several reports have correlated thermogenic phenotypes to *Ckmt1* or *Ckmt2* mRNA or protein expression [19,36, 37, 38, 39]. However, expression levels do not provide information regarding metabolic pathway activity. Aside from *Ckb* [3, 4, 5, 6,8], no published studies have demonstrated a functional role for any other CK isoform in murine adipocytes. Germline *Ckmt1* knockout mice have been used to study the FCC; however, no evidence of *Ckmt1* deletion was presented at the mRNA, protein, or functional level in bulk iWAT. [40]. Our results call the conclusions of this study into question. Not only is CKB the predominant isoform in mouse adipocytes, demonstrated by genetic deletion, CK activity assays, and functional studies, but single-cell RNA-seq data further show that *Ckmt1* is not a major isoform in non-parenchymal adipose cells either. Thus, given the lack of evidence for its expression in relevant adipose cell types in mice, conclusions based on *Ckmt1* expression in adipose tissue or the use of *Ckmt1*^−/−^ mice [40] should be interpreted with caution, especially when they overlook the established role of CKB [41, 42, 43].

Human white adipocytes express both CKB and CKMT2, where CKB was proposed to be purely cytosolic and CKMT2 was mitochondrial [44]. Interestingly, mitochondrial CK activity was partially retained following *CKMT2* knockdown, suggesting either incomplete silencing or compensation by mitochondrial CKB activity [44]. *CKB* knockdown increased the phosphocreatine-to-creatine ratio, suggesting a role in the reverse reaction (PCr+ADP→ATP+Cr). A reduction in this reverse activity may compromise ATP free energy maintenance, potentially driving a compensatory increase in respiration to preserve ATP levels. Indeed, this manipulation increased respiration; however, this respiratory effect persisted in the presence of a protonophore, indicating a mechanism independent of ATP-coupled respiration and a more complex role for CKB in human white adipocyte bioenergetics. Moreover, CKB-dependent modulation of the phosphocreatine-to-creatine ratio has been linked to the inflammatory status of white adipocytes, further underscoring its functional significance in these non-thermogenic cells [44]. This observation contrasts with findings in mouse brown adipocytes, and our findings herein in beige adipocytes, where *Ckb* knockdown reduces FCC-driven thermogenesis [4,5]. While CKB typically forms homodimers, it can also heterodimerize with creatine kinase M (CKM), producing the CK isoenzymes BB, MM, and MB [23,45]. Given that human white and brown adipocytes express CKB, CKMT1, and CKMT2 [22,44,46], and that the four arginine residues required for mitochondrial targeting of murine CKB are conserved in the human ortholog [4], it is intriguing to speculate that distinct mitochondrial CK isoform complexes, potentially including mitochondrial CKB, may exist in human adipocytes to support specialized metabolic functions.

To overcome the limitations of *in vivo* and *in vitro* models, we employed an *ex vivo* approach using acutely isolated mature adipocytes. *In vivo* systems provide physiological context but suffer from cellular heterogeneity, especially in iWAT [27,47]. *In vitro* systems enable specific interrogation of adipocytes but often lack full differentiation and fail to recapitulate beige fat physiology. To overcome these challenges, we isolated mature inguinal adipocytes from mice acclimated to different temperatures, enabling a physiologically relevant, adipocyte-specific investigation of CKB function. Across all models, our findings consistently demonstrate that CKB is the principal regulator of creatine metabolism in mouse white, beige, and brown adipocytes, and that it drives FCC-dependent thermogenesis in beige fat.

Promoting the browning of white adipose tissue may hold therapeutic promise. Environmental stimuli like cold exposure [3,4,20,26,48] and genetic strategies, such as repression of ZFP423 [6,48,49], effectively induce beige adipogenesis and activate both UCP1-dependent and UCP1-independent thermogenic programs. Our *ex vivo* approach, which enriches for mature adipocytes, allowed direct evaluation of CKB-dependent activity in white and beige adipocytes. The sharp decrease in CK activity and mitochondrial respiration upon *Ckb* deletion or TNAP inhibition underscores the key role of CKB in FCC-dependent beige adipocyte thermogenesis.

## 4 Materials and methods

### 4.1 Animals

*Ckb*^fl/fl^ and *Ckb*^AdipoqCre^ mice were previously described [4]. Mouse experiments were performed according to procedures approved by the Animal Resource Centre at McGill University and complied with guidelines set by the Canadian Council of Animal Care. The photoperiod was fixed at a 12-h light/12-h dark schedule (light 07:00 to 19:00) with lights on at 07:00 h being defined as Zeitgeber time 0 (ZT0). Mice had *ad libitum* access to drinking water and a chow diet (3.1 kcal/g energy density) with 24%, 16%, and 60% of Calories from protein, fat, and carbohydrate, respectively (2920X, Envigo, Madison, WI, USA). All mice were born and housed in groups (3–5 mice per cage) at 21 °C ± 1 °C with bedding and shredded paper strips in the cage until experimental intervention. For cold exposure experiments, mice were housed in cages placed in rodent incubators (Powers Scientific) with bedding and with *ad libitum* access to drinking water and chow diet (3–5 mice per cage). Mouse experiments used age-matched littermates and were conducted at the temperature indicated in each figure legend. Mice were euthanized by cervical dislocation and tissues were either immediately flash-frozen in liquid nitrogen and stored at −80 °C until further analysis, or processed immediately for isolation of mature adipocytes.

### 4.2 RNA extraction

Total RNA was extracted from frozen tissue or cultured adipocytes using TRIzol (Ambion, Life Technologies, cat. No. 15596018) and purified with RNeasy Mini spin columns (Qiagen, cat. No. 74106) according to the manufacturer's instructions. RNA was quantified using a NanoDrop 8000 Spectrophotometer (Thermo Scientific Pierce, Waltham, Maine, USA).

### 4.3 Reverse transcription quantitative polymerase chain reaction (RT-qPCR)

Purified RNA (1,000–2,000 ng) was reverse-transcribed using a High-Capacity cDNA reverse transcription kit (Applied Biosystems, cat. No. 4368814). The resultant cDNA was analysed by RT-qPCR. In brief, 20 ng of cDNA and 187.5 nmol of each primer were mixed with GoTaq qPCR Master Mix (Promega, cat. No. A6002) and RT-qPCR was performed in a 384-well format using a CFX384 Real-time PCR system (Bio-rad). Normalized mRNA expression was calculated using the ΔΔCt method, using *Ppib* mRNA as the reference gene. CFX Maestro 2017 was used for data collection. Primer sequences used for RT-qPCR were as follows: *Ppib* (forward, GGAGATGGCACAGGAGGAA; reverse, GCCCGTAGTGCTTCAGCTT) *Ckb* (forward, GCCTCACTCAGATCGAAACTC; reverse, GGCATGTGAGGATGTAGCCC); *Ckm* (forward, CTGACCCCTGACCTCTACAAT; reverse, CATGGCGGTCCTGGATGAT); *Ckmt1* (forward, TGAGGAGACCTATGAGGTATTTGC; reverse, TCATCAAAGTAGCCAGAACGGA); *Ckmt2* (forward, AGCAGGATCAGCAACGTCTC; reverse, TAATTATGCCAGATTCCCCTGG); *Alpl* (forward, CCAACTCTTTTGTGCCAGAGA; reverse, GGCTACATTGGTGTTGAGCTTTT); *Ucp1* (forward, ACTGCCACACCTCCAGTCATT; reverse, CTTTGCCTCACTCAGGATTGG); *Prdm16* (forward, CAGCACGGTGAAGCCATTC; reverse, GCGTGCATCCGCTTGTG); *Pparg2* (forward, TGCCTATGAGCACTTCACAAGAAAT; reverse, CGAAGTTGGTGGGCCAGAA).

### 4.4 Western blotting

Samples were prepared in lysis buffer (50 mM Tris, pH 7.4, 500 mM NaCl, 1% NP40, 20% glycerol, 5 mM EDTA and 1 mM phenylmethylsulphonyl fluoride (PMSF)), supplemented with a cocktail of protease inhibitors (Roche, 11836170001). The homogenates were centrifuged at 16,000 g for 10 min at 4 °C, and the supernatants were used for subsequent analyses. Protein concentration was determined using Pierce BCA Protein Assay Kit (Thermo Scientific, 23225). The quantity of protein lysate to use for each antibody was determined empirically. Protein lysates were denatured in Laemmli buffer (60 mM Tris, pH 6.8, 2% SDS, 10% glycerol, 0.05% bromophenol blue, 0.7 M β-mercaptoethanol), resolved by 10% Tris/Glycine SDS–PAGE and transferred to a polyvinylidene difluoride (PVDF) membrane. Primary antibodies were diluted in TBS containing 0.05% Tween (TBS-T), 5% BSA and 0.02% NaN3. Membranes were incubated overnight with primary antibodies at 4 °C. Secondary antibodies were diluted in TBS-T containing 5% milk for 45 min. Results were visualized with enhanced chemiluminescence Western blotting substrates (Bio-Rad) using a Chemidoc Imaging System (Bio-Rad). Dilutions for antibodies were as follows: VCL (Cell Signaling; cat. no. 13901; clone E1E9V; dilution: 1:5,000); UCP1 (Abclonal; cat. no. A7236; dilution: 1:10,000); CKB (Abclonal; cat. no. A12631; dilution: 1:1,000); TNAP (R&D; cat. no. AF2910; dilution: 1:200); PLIN1 (Cell Signaling; cat. no. 9349; dilution: 1:1,000); Anti-rabbit (Promega, cat. no. W4011; dilution: 1:10,000); Anti-goat (Promega, cat. no. V805A; dilution: 1:10,000). Western blots were quantified using ImageJ [49].

### 4.5 Creatine kinase (CK) activity

A coupled enzymatic reaction (pyruvate kinase and lactate dehydrogenase) was used to determine creatine kinase activity in the forward direction (*creatine + ATP* → *ADP + phosphocreatine*). Absorbance at 340 nM was measured to determine the NADH oxidation rate using a BioTek Synergy H1 plate reader in kinetic mode. The assay was performed at 25 °C by supplementing assay buffer (20 mM MgCl_2_, 100 mM KCl, 5 μΜ oligomycin, and 50 mM Tris pH 9.0) with coupling substrates (5 mM ATP, 4 mM PEP, and 0.45 mM NADH), 40 μg of protein, and 10 mM creatine.

### 4.6 Metabolite sample preparation of brown and white adipose tissue

Mice were injected intraperitoneally with d3-creatine (Cambridge Isotope Laboratories, DLM-1302) at 2 mg/kg or 4 mg/kg body weight. After 4 h, mice were euthanized with cervical dislocation and tissues were harvested and flash-frozen in liquid nitrogen until further analysis. Frozen tissues were crushed to fine powder in liquid nitrogen cooled mortar and pestle and quantitatively weighted into a fresh 2 ml tube. Into the pre-chilled 2 ml tube containing the pulverized frozen tissue sample (powder), 1,140 μl of 50% MeOH/50% LC/MS water solution (at −20 °C or colder), was added along with 4 ceramic beads (2.8 mm). Samples were vortexed for 10 s, 660 μl ice cold acetonitrile was added and vortexed again for another 10 s. Samples were then homogenized in bead beater homogenizer (Qiagen TissueLyser) for 2 min at 30 Hz, up to 4 times until tissue was homogenously lysed. Homogenate was transferred into a fresh 5 ml tube, and 1,800 μl of ice-cold dichloromethane and 900 μl of ice-cold H_2_O were added before vortexing for 1 min. Upon letting the mixture partition on ice for 10 min, samples were spun down for 10 min at 4,000 rpm set at 1 °C. The upper aqueous phase containing the water-soluble metabolites was transferred into to a fresh tube on dry ice and dried using a chilled vacuum centrifuge set to 4 °C or cooler, overnight (CentriVap Centrifugal Concentrator, Labconco).

### 4.7 Metabolite analysis by mass spectrometry

Metabolites were profiled at the Rosalind and Morris Goodman Cancer Institute Metabolomics Innovation Resource at McGill University. For targeted metabolite analysis, samples were injected onto an Agilent 6470 Triple Quadrupole (QQQ)–LC–MS/MS (Agilent Santa Clara, CA). Chromatographic separation of metabolites was achieved by using a 1290 Infinity ultra-performance quaternary pump liquid chromatography system (Agilent Santa Clara, CA). Compounds were chromatographically separated using a Scherzo SM-C18 column 3 μm, 3.0 × 150 mm (Imtakt Corp, JAPAN) maintained at 10 °C. The chromatographic gradient started at 100% mobile phase A (5 mM ammonium acetate in water) with a 5 min gradient to 100% B (200 mM ammonium acetate in 20% ACN/80% water) at a flow rate of 0.4 ml/min. This was followed by a 5 min hold time at 100% mobile phase B and a subsequent re-equilibration time (6 min) before next injection. A sample temperature was maintained at 4 °C before the next injection of 5 ml of sample. Eluting compounds were ionized using an Agilent dual a Jet Stream™ electrospray ionization source, and samples were analyzed in positive mode. The source was set with the following parameters: Gas temperature and flow of 300 °C and 5 l/min respectively, nebulizer pressure of 45 psi and capillary voltage of 3,500 V. Multiple reaction monitoring parameters (qualifier/quantifier ions and retention times) were optimized using authentic metabolite standards. Data were collected on matched samples that were treated with unlabeled creatine to separate deuterium incorporation from interfering ions. Stable isotope tracer data were analyzed using MassHunter Profinder Software B.08.00 (Agilent Technologies), which includes matrix correction, or data were analyzed using area under the curve from MassHunter Quant (Agilent Technologies). We used external calibration curves prepared in the same reconstitution solvent as the samples to ensure analytes fell within the instrument's linear dynamic range. If sample signals exceeded this range, dilution was performed. In this study, due to high creatine abundance, samples were diluted 1,000-fold in Figure 2. In contrast, samples from a different experiment in Fig. S1 did not require dilution. Since, in electrospray ionization, signal intensity is not linearly proportional to concentration [50], scaling up a diluted sample's signal by the dilution factor does not yield a valid comparison to undiluted samples. Additionally, samples in the pilot study of Fig. S1 were resuspended in 50 μl, but for the main experiment in Figure 2, resuspension was done in 30 μl to concentrate the target metabolites. Importantly, in Figure 2, *Ckb*^fl/fl^ and *Ckb*^AdipoqCre^ samples were treated identically ensuring appropriate comparability.

### 4.8 Isolation and differentiation of white preadipocytes *in vitro*

Inguinal white fat from 10-day-old mice (4–5 mice per prep) was excised, minced with spring scissors, and digested with digestion buffer containing 10 mg/ml Collagenase D, 3U/ml Dispase II, and 10 mM CaCl_2_ in 1X PBS with continuous shaking at 37 °C for 45 min angled at 45°. The digestion mixture was vortexed twice every 5 min for 2 s. After shaking, 20 ml of DMEM/F12 GlutaMAX (Gibco; 10565-042), supplemented with 10% FBS (Sigma, Cat. No. F1051) and 1% Penicillin/Streptomycin was added and the solution was filtered through a 100 μM cell strainer into a fresh falcon tube and spun at 600 g for 5 min at 4 °C (Sorvall Legend RT + Centrifuge; Thermo Scientific), with acceleration and deceleration set to 9 (maximum). Floating lipids and the remaining media were aspirated off, leaving the pelleted stromal vascular fraction (SVF) intact. The SVF pellet was re-suspended in complete media before filtering through a 40 μM cell strainer and plating onto a 10 cm TC-treated cell culture dish (Corning). The following day, once the cells attached to the dish, dead cells and cell debris were shaken off and washed with complete media up to 4 times. Media was changed every day and cells were passaged at 60–70% confluency until they were seeded onto 6-cm cell culture plates (Corning), after which were grown to post-confluency (∼1 × 10^6^ cells) to start the differentiation protocol. White adipocytes were differentiated over 7 days; culturing in DMEM/F12 GlutaMAX, 10% FBS and 1% Penicillin/Streptomycin, supplemented with differentiation cocktail (1 μM rosiglitazone, 0.5 mM IBMX, 1 μM dexamethasone and 870 nM insulin) on days 0–2 and supplemented with maintenance cocktail (1 μM rosiglitazone and 870 nM insulin) on days 2–7. Media was changed every 2 days during differentiation.

### 4.9 Metabolite sample preparation of beige adipocytes cultured *in vitro*

On day 7 of differentiation, adipocytes were supplemented with 150 μM D3-creatine (Cambridge Isotope Laboratories, DLM-1302) in maintenance cocktail (described above) containing dialyzed FBS for 4 h. Post-incubation, adipocytes were scraped off the plate in 350 μl of methanol/HPLC-grade water (50:50 (v/v), 0.22 ml of ice-cold acetonitrile (ACN) (at −20 °C or colder) and six ceramic beads (1.4 mm diameter). The mixture was homogenized with bead beating (QiagenTissueLyser) for 2 min at 30 Hz, after which, 0.6 ml of ice-cold dichloromethane and 0.3 ml of ice-cold HPLC-grade water were added. Samples were then vortexed for 1 min, incubated on ice for 10 min and centrifuged at 1,500 g for 10 min at 1 °C. Water-soluble metabolites in the upper polar phase were collected and dried using a chilled vacuum centrifuge operating at a sample temperature of 4 °C (CentriVap Centrifugal Concentrator, Labconco). Dried metabolites were analyzed in a manner described in Method 4.7.

### 4.10 Isolation of mature white and beige adipocytes

Inguinal white adipose tissue (iWAT), from cold (4 °C ± 1 °C)-acclimated mice (3 weeks of acclimation), was minced and digested in a Krebs–Ringer bicarbonate modified buffer (KRBMB: 135 mM NaCl; 5 mM KCl; 1 mM CaCl_2_; 1 mM MgCl_2_; 0.4 mM K_2_HPO_4_; 25 mM NaHCO_3_; 20 mM HEPES; 10 mM glucose; 4% fatty acid-free BSA), supplemented with 2 mg/ml collagenase B (Worthington) and 1 mg/ml soybean trypsin inhibitor (Worthington) with continuous shaking at 150 rpm at 37 °C for 50 min, angled at 45°. The tissue suspension was filtered through a 100 μm cell strainer into a 50 ml tube containing 20 ml of 3% fatty acid-free BSA/PBS supplemented with 4 mM EDTA (pH = 8.0) and gently mixed by gently inverting the tube 3–4 times. Adipocytes were allowed to float for 40 min at room temperature before the top fat layer (where mature adipocytes reside) was removed with a 2 ml plastic Pasteur pipette into a 15 ml tube containing 10 ml of DMEM/F12 GlutaMAX (Invitrogen; 10565-042), supplemented with 10% FBS (Sigma, Cat. No. F1051). Adipocytes within the transferred fat layer were then gently mixed by inverting the tube three times and allowed to float for an additional 40 min before transferring the fat layer (containing the adipocytes) into a fresh tube containing 3 ml of DMEM/F12 GlutaMAX/10% FBS. Cell numbers were determined using the method described below. Any residual mature adipocytes that remained after the initial transfer from the 50 ml to the 15 ml tube were aspirated prior to centrifuging the sample for 5 min at 200 g to pellet the SVF.

### 4.11 Assessment of mature adipocyte cell concentration

A 100 μl aliquot of floated adipocyte sample was moved into separate 5 ml tube and mixed with 2 ml of Nuclear Lysis Buffer (250 mM Sucrose; 10 mM KCl; 1.5 mM MgCl2; 0.1% IGEPAL). This lysis mixture was incubated at room temperature for 10 min and vortexed every 3 min. After the lysis of the cells, nuclei were spun down in swing bucket centrifuge set to 1,000 g for 10 min followed by the removal of the supernatant and resuspending the nuclei pellet in 100 ul of Trypan Blue/Nuclear Lysis Buffer mixture (1:1). Nuclei number was determined using a Bright-Line Hemacytometer (Hausser Scientific).

### 4.12 Mitochondrial isolation from iWAT

iWAT was excised from four mice, minced in 10 ml of MSH buffer (210 mM mannitol; 70 mM sucrose; 20 mM HEPES, pH 7.8, and 2 mg/ml fatty acid-free BSA), and homogenized on ice with a mechanical Potter-Elvehjem homogenizer (15 strokes). The homogenate was spun at 8,500 g for 10 min at 4 °C and the fat layer was discarded. The pellet was resuspended in MSH buffer and spun at 600 g for 5 min at 4 °C to pellet the nuclei and cell debris and the post-nuclear supernatant was spun at 8,500 g for 10 min at 4 °C to pellet the mitochondria. Mitochondrial pellet was resuspended in storage buffer (100 mM KCl and 20 mM K-TES pH 7.2) and the total protein was quantified using the Pierce BCA Protein Assay Kit (Thermo Scientific, 23225).

### 4.13 Respirometry of purified iWAT mitochondria using an oxygen electrode

A Clark-type electrode was used to determine the quantitative contribution of futile creatine cycling in beige adipocyte mitochondria isolated from the iWAT of cold-acclimated mice. Mitochondria (0.150 mg) were added to respiration buffer (125 mM sucrose, 20 mM K-TES, pH 7.2, 2 mM MgCl_2_, 1 mM EDTA, 0.5 mM KH_2_PO_4_, 4% fatty acid-free BSA, 10 mM pyruvate, and 5 mM malate) supplemented with 3 mM GDP. The total volume in the respiration chamber was 0.7 ml, and 60 nmol (100 μM) ADP was added to initiate ADP-dependent respiration. SBI-425 was used at a final concentration of 10 μM. All reported respiratory rates were normalized to the state 2 respiratory rate (defined as the mitochondrial oxygen consumption rate prior to ADP addition). Rank Brothers Dual Digital model 20: Picolog 6 data logging software was used for data collection.

### 4.14 Single cell RNA sequencing data analysis

Data was obtained from publicly available repositories.

1) GSE227441 [34]**:** scRNA-seq gene expression in stromal vascular cells from iWAT. Raw data was processed, integrated and clustered using the pipeline shared by the authors, which is available at: https://github.com/calhounr/Aging-impairs-cold-induced-beige-adipogenesis-and-adipocyte-metabolic-reprogramming
2) GSE154047 [51]**:** scRNA-seq gene expression in stromal vascular cells from iWAT. Data was processed, integrated and clustered using the same method as in Fig. 3B.
3) GSE160585 [52]**:** scRNA-seq data of stromal vascular cells from iBAT. Processed data in the form of a SeuratObject was obtained directly from the authors of the original publication.

### 4.15 Statistical analysis

Statistical analyses were performed with GraphPad Prism 10. Data analyses were performed using Microsoft Office Excel (v.16.56). Data are expressed as the mean ± s.e.m. Tests for normal distribution were performed by Shapiro–Wilk test. We used unpaired two-tailed Student's *t*-test for pairwise comparisons and one- and two-way ANOVA for multiple comparisons involving two independent variables, with *P* < 0.05 considered statistically significant. The specific *n* values noted in each figure legend represent independent biological replicates.

## CRediT authorship contribution statement

**Jakub Bunk:** Writing – review & editing, Writing – original draft, Validation, Methodology, Investigation, Formal analysis, Conceptualization. **Mina Ersin:** Writing – review & editing, Validation, Methodology, Investigation, Formal analysis. **Mohammed F. Hussain:** Investigation, Formal analysis. **Bozena Samborska:** Writing – review & editing, Validation, Investigation, Formal analysis. **Maria Guerra-Martinez:** Investigation. **Drishti Soni:** Investigation. **Lawrence Kazak:** Writing – review & editing, Writing – original draft, Supervision, Project administration, Funding acquisition, Conceptualization.

## Data statement

All the data acquired are presented in the figures.

## Declaration of competing interest

The authors declare the following financial interests/personal relationships which may be considered as potential competing interests:

Lawrence Kazak reports financial support provided by Canadian Institutes of Health Research. Lawrence Kazak reports financial support provided by Natural Sciences and Engineering Research Council of Canada. Jakub Bunk reports financial support provided by Quebec Health Research Fund. Mohammed Faiz Hussain reports financial support provided by Natural Sciences and Engineering Research Council of Canada. Maria Guerra-Martinez reports financial support provided by Quebec Health Research Fund. If there are other authors, they declare that they have no known competing financial interests or personal relationships that could have appeared to influence the work reported in this paper.

## Data Availability

Data will be made available on request.

## References

[1] Wang T., Sharma A.K., Wu C., Maushart C.I., Ghosh A., Yang W. (2024). Single-nucleus transcriptomics identifies separate classes of UCP1 and futile cycle adipocytes. Cell Metab.

[2] Vargas-Castillo A., Sun Y., Smythers A.L., Grauvogel L., Dumesic P.A., Emont M.P. (2024). Development of a functional beige fat cell line uncovers independent subclasses of cells expressing UCP1 and the futile creatine cycle. Cell Metab.

[3] Rahbani J.F., Bunk J., Lagarde D., Samborska B., Roesler A., Xiao H. (2024). Parallel control of cold-triggered adipocyte thermogenesis by UCP1 and CKB. Cell Metab.

[4] Rahbani J.F., Roesler A., Hussain M.F., Samborska B., Dykstra C.B., Tsai L. (2021). Creatine kinase B controls futile creatine cycling in thermogenic fat. Nature.

[5] Rahbani J.F., Scholtes C., Lagarde D.M., Hussain M.F., Roesler A., Dykstra C.B. (2022). ADRA1A-Galphaq signalling potentiates adipocyte thermogenesis through CKB and TNAP. Nat Metab.

[6] Hepler C., Weidemann B.J., Waldeck N.J., Marcheva B., Cedernaes J., Thorne A.K. (2022). Time-restricted feeding mitigates obesity through adipocyte thermogenesis. Science.

[7] Duerre D.J., Hansen J.K., John S.V., Jen A., Carrillo N.D., Bui H. (2025). Haem biosynthesis regulates BCAA catabolism and thermogenesis in brown adipose tissue. Nat Metab.

[8] Bunk J., Hussain M.F., Delgado-Martin M., Samborska B., Ersin M., Shaw A. (2025). The Futile Creatine Cycle powers UCP1-independent thermogenesis in classical BAT. Nat Commun.

[9] Oeckl J., Janovska P., Adamcova K., Bardova K., Brunner S., Dieckmann S. (2022). Loss of UCP1 function augments recruitment of futile lipid cycling for thermogenesis in murine brown fat. Mol Metab.

[10] Ikeda K., Kang Q., Yoneshiro T., Camporez J.P., Maki H., Homma M. (2017). UCP1-independent signaling involving SERCA2b-mediated calcium cycling regulates beige fat thermogenesis and systemic glucose homeostasis. Nat Med.

[11] Granneman J.G., Burnazi M., Zhu Z., Schwamb L.A. (2003). White adipose tissue contributes to UCP1-independent thermogenesis. Am J Physiol Endocrinol Metab.

[12] Ukropec J., Anunciado R.P., Ravussin Y., Hulver M.W., Kozak L.P. (2006). UCP1-independent thermogenesis in white adipose tissue of cold-acclimated Ucp1-/- mice. J Biol Chem.

[13] Ukropec J., Anunciado R.V., Ravussin Y., Kozak L.P. (2006). Leptin is required for uncoupling protein-1-independent thermogenesis during cold stress. Endocrinology.

[14] Wu J., Bostrom P., Sparks L.M., Ye L., Choi J.H., Giang A.H. (2012). Beige adipocytes are a distinct type of thermogenic fat cell in mouse and human. Cell.

[15] Young P., Arch J.R., Ashwell M. (1984). Brown adipose tissue in the parametrial fat pad of the mouse. FEBS Lett.

[16] Xue R., Lynes M.D., Dreyfuss J.M., Shamsi F., Schulz T.J., Zhang H. (2015). Clonal analyses and gene profiling identify genetic biomarkers of the thermogenic potential of human brown and white preadipocytes. Nat Med.

[17] Shinoda K., Luijten I.H., Hasegawa Y., Hong H., Sonne S.B., Kim M. (2015). Genetic and functional characterization of clonally derived adult human brown adipocytes. Nat Med.

[18] Seale P., Bjork B., Yang W., Kajimura S., Chin S., Kuang S. (2008). PRDM16 controls a brown fat/skeletal muscle switch. Nature.

[19] Bertholet A.M., Kazak L., Chouchani E.T., Bogaczynska M.G., Paranjpe I., Wainwright G.L. (2017). Mitochondrial Patch Clamp of Beige Adipocytes Reveals UCP1-Positive and UCP1-Negative Cells Both Exhibiting Futile Creatine Cycling. Cell Metab.

[20] Kazak L., Chouchani E.T., Jedrychowski M.P., Erickson B.K., Shinoda K., Cohen P. (2015). A Creatine-Driven Substrate Cycle Enhances Energy Expenditure and Thermogenesis in Beige Fat. Cell.

[21] Liu J., Cheng Y., Liu Q., Long Q., Liang S., Sun W. (2025). LETM-domain containing 1 (LETMD1) protects against obesity via enhancing UCP1-independent energy expenditure in human beige adipocytes. Theranostics.

[22] Muller S., Balaz M., Stefanicka P., Varga L., Amri E.Z., Ukropec J. (2016). Proteomic Analysis of Human Brown Adipose Tissue Reveals Utilization of Coupled and Uncoupled Energy Expenditure Pathways. Sci Rep.

[23] Wallimann T., Wyss M., Brdiczka D., Nicolay K., Eppenberger H.M. (1992). Intracellular compartmentation, structure and function of creatine kinase isoenzymes in tissues with high and fluctuating energy demands: the 'phosphocreatine circuit' for cellular energy homeostasis. Biochem J.

[24] Kazak L., Cohen P. (2020). Creatine metabolism: energy homeostasis, immunity and cancer biology. Nat Rev Endocrinol.

[25] Samborska B., Roy D.G., Rahbani J.F., Hussain M.F., Ma E.H., Jones R.G. (2022). Creatine transport and creatine kinase activity is required for CD8(+) T cell immunity. Cell Rep.

[26] Sun Y., Rahbani J.F., Jedrychowski M.P., Riley C.L., Vidoni S., Bogoslavski D. (2021). Mitochondrial TNAP controls thermogenesis by hydrolysis of phosphocreatine. Nature.

[27] Roh H.C., Tsai L.T., Lyubetskaya A., Tenen D., Kumari M., Rosen E.D. (2017). Simultaneous Transcriptional and Epigenomic Profiling from Specific Cell Types within Heterogeneous Tissues In Vivo. Cell Rep.

[28] Wimer L., Goncharova E., Galkina S., Nyangau E., Shankaran M., Davis A. (2023). The D(3) -creatine dilution method non-invasively measures muscle mass in mice. Aging Cell.

[29] Roh H.C., Tsai L.T.Y., Shao M., Tenen D., Shen Y., Kumari M. (2018). Warming Induces Significant Reprogramming of Beige, but Not Brown, Adipocyte Cellular Identity. Cell Metab.

[30] Cypess A.M., Chen Y.C., Sze C., Wang K., English J., Chan O. (2012). Cold but not sympathomimetics activates human brown adipose tissue in vivo. Proc Natl Acad Sci U S A.

[31] Chen Y., Ikeda K., Yoneshiro T., Scaramozza A., Tajima K., Wang Q. (2019). Thermal stress induces glycolytic beige fat formation via a myogenic state. Nature.

[32] Chi J., Wu Z., Choi C.H.J., Nguyen L., Tegegne S., Ackerman S.E. (2018). Three-Dimensional Adipose Tissue Imaging Reveals Regional Variation in Beige Fat Biogenesis and PRDM16-Dependent Sympathetic Neurite Density. Cell Metab.

[33] Shao M., Wang Q.A., Song A., Vishvanath L., Busbuso N.C., Scherer P.E. (2019). Cellular Origins of Beige Fat Cells Revisited. Diabetes.

[34] Holman C.D., Sakers A.P., Calhoun R.P., Cheng L., Fein E.C., Jacobs C. (2024). Aging impairs cold-induced beige adipogenesis and adipocyte metabolic reprogramming. Elife.

[35] Rahbani J.F., Chouchani E.T., Spiegelman B.M., Kazak L. (2022). Measurement of Futile Creatine Cycling Using Respirometry. Methods Mol Biol.

[36] Pydi S.P., Jain S., Tung W., Cui Y., Zhu L., Sakamoto W. (2019). Adipocyte beta-arrestin-2 is essential for maintaining whole body glucose and energy homeostasis. Nat Commun.

[37] Wang L., Pydi S.P., Cui Y., Zhu L., Meister J., Gavrilova O. (2019). Selective activation of G(s) signaling in adipocytes causes striking metabolic improvements in mice. Mol Metab.

[38] Pollard A.E., Martins L., Muckett P.J., Khadayate S., Bornot A., Clausen M. (2019). AMPK activation protects against diet induced obesity through Ucp1-independent thermogenesis in subcutaneous white adipose tissue. Nat Metab.

[39] Maurer S.F., Fromme T., Mocek S., Zimmermann A., Klingenspor M. (2020). Uncoupling protein 1 and the capacity for nonshivering thermogenesis are components of the glucose homeostatic system. Am J Physiol Endocrinol Metab.

[40] Politis-Barber V., Petrick H.L., Raajendiran A., DesOrmeaux G.J., Brunetta H.S., Dos Reis L.M. (2022). Ckmt1 is Dispensable for Mitochondrial Bioenergetics Within White/Beige Adipose Tissue. Function (Oxf).

[41] Politis-Barber V., Petrick H.L., Raajendiran A., DesOrmeaux G.J., Brunetta H.S., dos Reis L.M. (2022). Ckmt1 is Dispensable for Mitochondrial Bioenergetics Within White/Beige Adipose Tissue. Function.

[42] Paulo E., Zhang Y., Masand R., Huynh T.L., Seo Y., Swaney D.L. (2021). Brown adipocyte ATF4 activation improves thermoregulation and systemic metabolism. Cell Rep.

[43] Yu X., Chen S., Funcke J.B., Straub L.G., Pirro V., Emont M.P. (2025). The GIP receptor activates futile calcium cycling in white adipose tissue to increase energy expenditure and drive weight loss in mice. Cell Metab.

[44] Maqdasy S., Lecoutre S., Renzi G., Frendo-Cumbo S., Rizo-Roca D., Moritz T. (2022). Impaired phosphocreatine metabolism in white adipocytes promotes inflammation. Nat Metab.

[45] Eppenberger H.M., Dawson D.M., Kaplan N.O. (1967). The comparative enzymology of creatine kinases. I. Isolation and characterization from chicken and rabbit tissues. J Biol Chem.

[46] Cero C., Shu W., Reese A.L., Douglas D., Maddox M., Singh A.P. (2023). Standardized in vitro Models of Human Adipose Tissue Reveal Metabolic Flexibility in Brown Adipocyte Thermogenesis. Endocrinology.

[47] Kwok K.H.M., Lam K.S.L., Xu A. (2016). Heterogeneity of white adipose tissue: molecular basis and clinical implications. Experimental & Molecular Medicine.

[48] Shabalina I.G., Petrovic N., de Jong J.M., Kalinovich A.V., Cannon B., Nedergaard J. (2013). UCP1 in brite/beige adipose tissue mitochondria is functionally thermogenic. Cell Rep.

[49] Schneider C.A., Rasband W.S., Eliceiri K.W. (2012). NIH Image to ImageJ: 25 years of image analysis. Nat Methods.

[50] Stahnke H., Kittlaus S., Kempe G., Alder L. (2012). Reduction of matrix effects in liquid chromatography-electrospray ionization-mass spectrometry by dilution of the sample extracts: how much dilution is needed?. Anal Chem.

[51] Henriques F., Bedard A.H., Guilherme A., Kelly M., Chi J., Zhang P. (2020). Single-Cell RNA Profiling Reveals Adipocyte to Macrophage Signaling Sufficient to Enhance Thermogenesis. Cell Rep.

[52] Shamsi F., Piper M., Ho L.L., Huang T.L., Gupta A., Streets A. (2021). Vascular smooth muscle-derived Trpv1(+) progenitors are a source of cold-induced thermogenic adipocytes. Nat Metab.

