# Supplementary material for: Creatine kinase B mediates UCP1-independent beige fat thermogenesis via the futile creatine cycle in mice
Source: Mol Metab. 2025 Jun 23;98:102193. doi: 10.1016/j.molmet.2025.102193 (PMC12271865; doi:10.1016/j.molmet.2025.102193)
Supplement: Multimedia component 1 [file mmc1.docx]

Supplementary Information

**Creatine kinase B mediates Ucp1-independent beige fat thermogenesis in mice.**

Jakub Bunk^1,2,3^, Mina Ersin^1,2,3^, Mohammed F. Hussain^1,2^, Bozena Samborska^1^, Maria Guerra-Martinez^1,2^, Drishti Soni^1,2^ and Lawrence Kazak^1,2,^*.

^1^Rosalind & Morris Goodman Cancer Institute, McGill University, Montreal, QC, H3A 1A3, Canada

^2^Department of Biochemistry, McGill University, Montreal, QC, H3G 1Y6, Canada

^3^These authors contributed equally to this work

Correspondence to: [lawrence.kazak@mcgill.ca](mailto:lawrence.kazak@mcgill.ca)

**
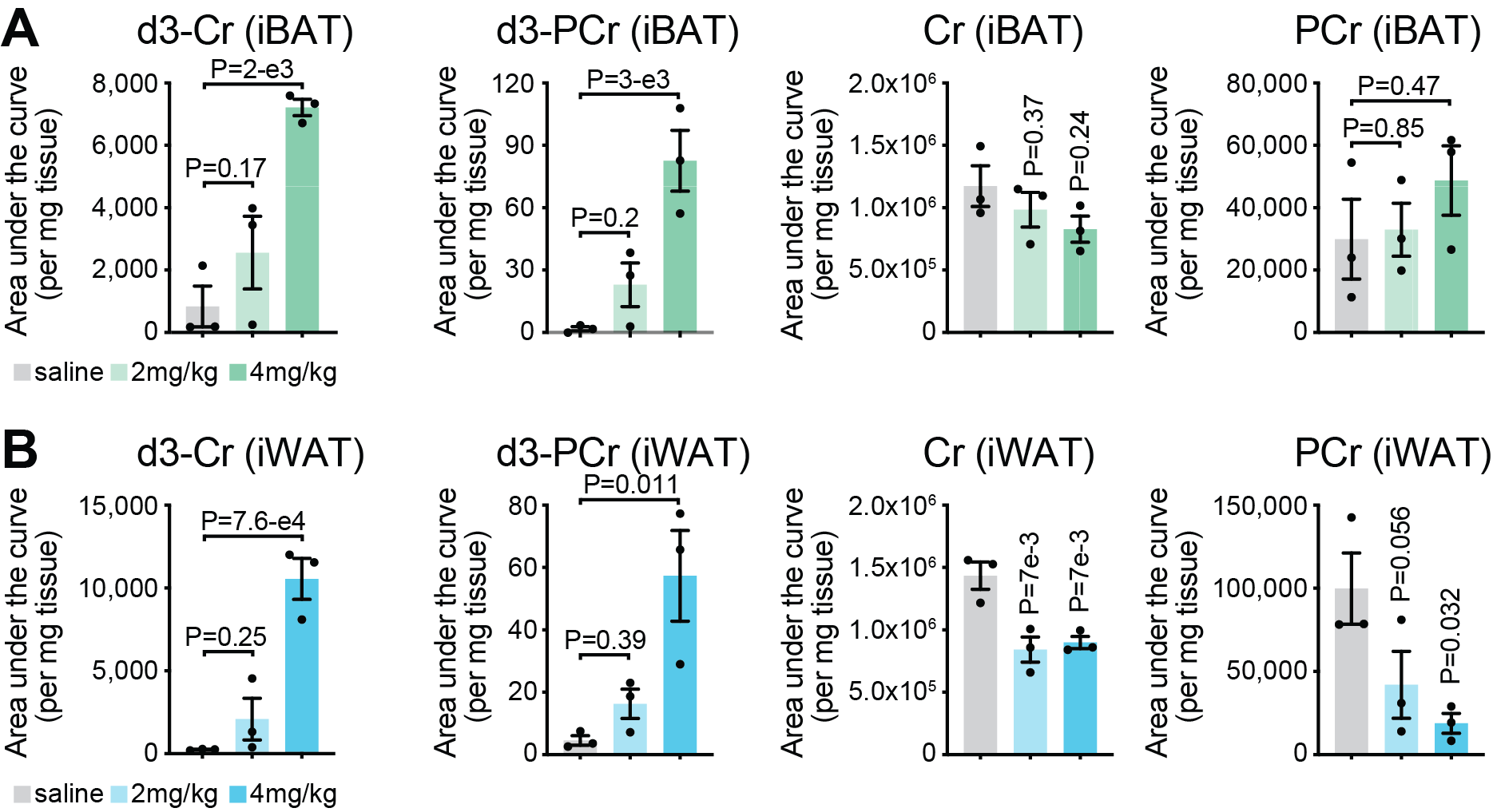
Supplemental Fig 1. PCr synthesis in interscapular brown adipose tissue and inguinal white adipose tissue *in vivo*. A,** LC-MS analysis of deuterated (m+3) creatine (d3-Cr), deuterated phosphocreatine (d3-PCr), unlabelled creatine (Cr), and unlabelled phosphocreatine (PCr) in iBAT of 10-week old *Ckb*^fl/fl^ mice 4 hours after injection with saline or d3-Cr (2 mg/kg or 4mg/kg) (*n* = 3 per group). **B,** LC-MS analysis of d3-Cr, d3-PCr, Cr, and PCr in iWAT of *Ckb*^fl/fl^ mice injected as in Supplemental Fig. 1A. Data are presented as mean ± s.e.m. and *n* numbers are of biologically independent experiments. *P* value were calculated using one-way ANOVA with Holm-Šídák's multiple comparisons test (**A-B**).

**
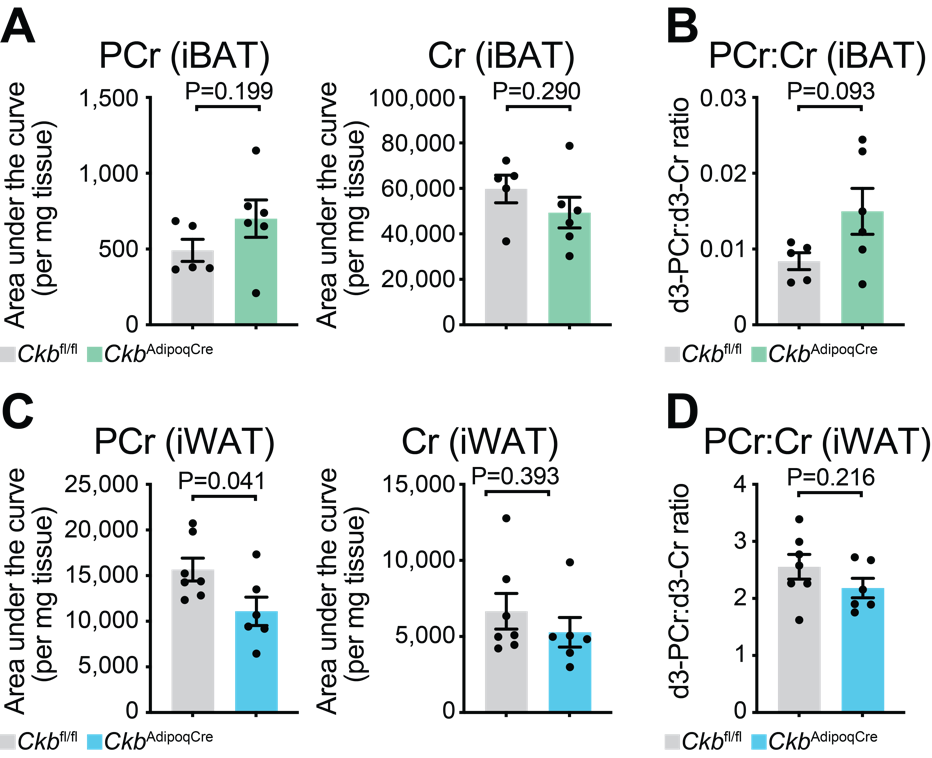
Supplemental Figure 2. Phosphocreatine and creatine levels in iBAT and iWAT of *Ckb*^fl/fl^ and *Ckb*^AdipoqCre^ male mice. A,** LC-MS analysis of unlabeled PCr and Cr levels in iBAT of *Ckb*^fl/fl^ and *Ckb^AdipoqCre^* mice (*n* = 5, 6 per group) 4 hours after injection with 4 mg/kg of d3-Cr. **B,** Ratio of PCr to Cr in samples from Supplemental Fig. 1A. **C,** LC-MS analysis of unlabeled PCr and Cr levels in iWAT of *Ckb*^fl/fl^ and *Ckb^AdipoqCre^* mice (*n* = 7, 6 per group) 4 hours after injection with 4 mg/kg of d3-Cr. **D,** Ratio of PCr to Cr in samples from Supplemental Fig. 1C. Data are presented as mean ± s.e.m. and *n* numbers are of biologically independent experiments. *P* value were calculated using unpaired t-test (**A-D**).

**
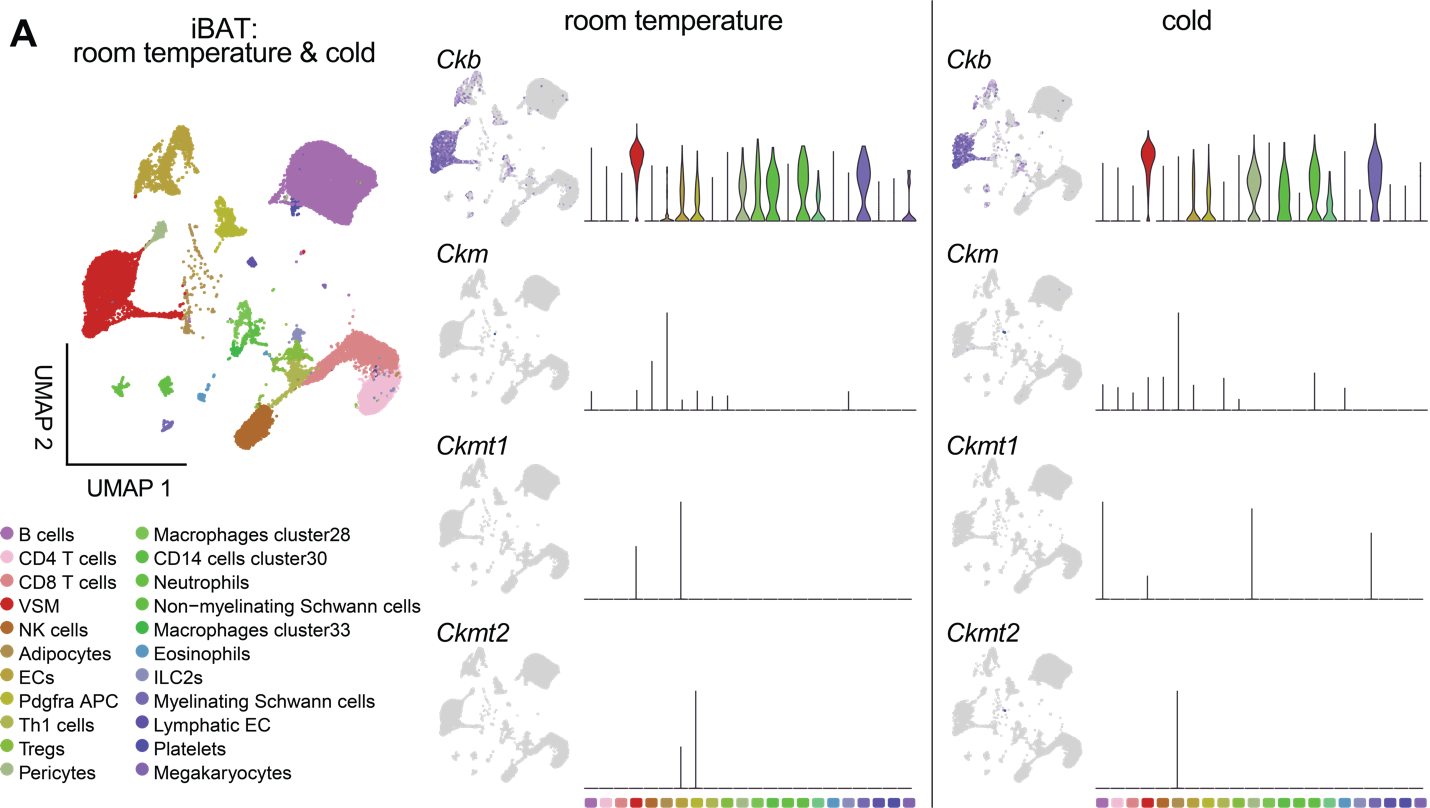
Supplemental Figure 3. Single cell RNA sequencing (scRNA-seq) analysis of iBAT stromal vascular cells. A,** scRNA-seq of stromal vascular cells obtained from [GSE160585](https://www.ncbi.nlm.nih.gov/geo/query/acc.cgi?acc=GSE160585). Integrated UMAP plot (left) with individual gene UMAP plots and violin plots (right) showing the expression levels of detected CKs.

**
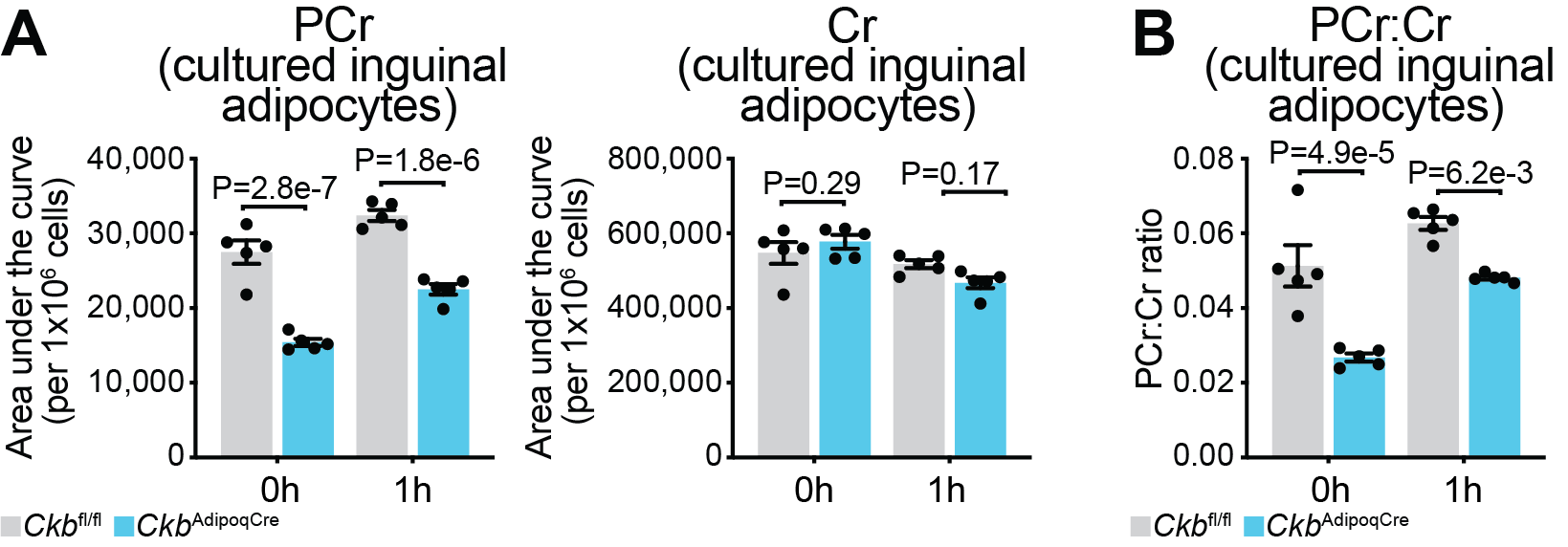
Supplemental Figure 4. Phosphocreatine and creatine levels in *Ckb*^fl/fl^ and *Ckb*^AdipoqCre^ cultured inguinal adipocytes. A-B,** Cultured inguinal adipocytes were incubated for 1 hour with 150 μM d3-Cr. **A,** LC-MS analysis of PCr (left) and Cr (right) levels in cultured *Ckb*^fl/fl^ and *Ckb*^AdipoqCre^ inguinal adipocytes (*n* = 5 per group). **B,** Ratio of PCr to Cr from Supplemental Fig 3A. Data are presented as mean ± s.e.m. and *n* numbers are of biologically independent experiments. *P* value were calculated using two-way ANOVA with Holm-Šídák's multiple comparisons test (**A-B**).

**
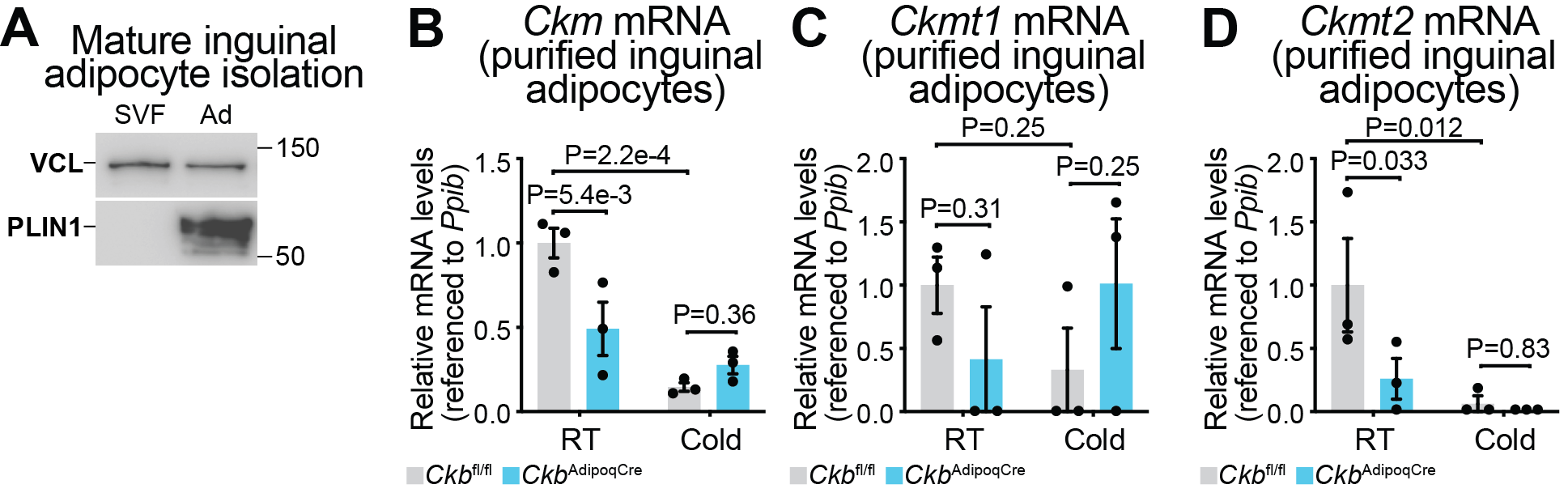
Supplemental Figure 5. CK isoform expression in purified inguinal adipocytes from RT- or cold-acclimated mice. A,** Western blot of PLIN1 in stromal vascular fraction and acutely isolated inguinal white adipocytes isolated from *Ckb*^fl/fl^ male mice housed at RT. VCL was used as a loading control. **B,** RT-qPCR from acutely isolated inguinal white adipocytes of 12-13-week-old *Ckb*^fl/fl^ and *Ckb^AdipoqCre^* male mice, exposed to RT or Cold for 3 weeks (*n* = 3 per group). Data are presented as mean ± s.e.m. and *n* numbers are of biologically independent experiments. *P* value were calculated using two-way ANOVA with Fisher LSD post-hoc test (**B-D**).
